# Promoting Social Participation and Recovery Using Virtual Reality–Based Interventions Among People With Mental Health and Substance Use Disorders: Qualitative Study

**DOI:** 10.2196/46136

**Published:** 2023-04-27

**Authors:** Jan Aasen, Kari Galaaen, Fredrik Nilsson, Torgeir Sørensen, Lars Lien, Marja Leonhardt

**Affiliations:** 1 Norwegian National Advisory Unit on Concurrent Substance Use and Mental Health Disorders Innlandet Hospital Trust Brummundal Norway; 2 VID Specialized University Oslo Norway; 3 RIO– a Norwegian users’ association in the field of alcohol and drugs Oslo Norway; 4 Department of Health and Social Science Inland Norway University of Applied Science Elverum Norway

**Keywords:** mental health disorders and substance use disorders, MHD, SUD, recovery, social participation, social functioning impairments, virtual reality–based interventions, VRI, reflexive thematic analysis, qualitative study

## Abstract

**Background:**

People with mental health disorders (MHDs) and substance use disorders (SUDs) are a highly vulnerable group, particularly affected by social exclusion, marginalization, and disconnectedness. Virtual reality technology holds a potential for simulating social environments and interactions to mitigate the social barriers and marginalization faced by people recovering from MHDs and SUDs. However, it is still unclear how we can harness the greater ecological validity of virtual reality–based interventions targeting social and functional impairments in individuals with MHDs and SUDs.

**Objective:**

The aim of this paper was to explore how service providers in community-based MHD and SUD health care services perceive the barriers to social participation among adults recovering from MHDs and SUDs to provide a broader understanding of how learning experiences can be modeled to promote social participation in virtual reality environments.

**Methods:**

Two semistructured, open-ended, and dual-moderator focus group interviews were conducted with participants representing different community-based MHD and SUD health care services. Service providers were recruited from their MHD and SUD services in our collaborating municipality in Eastern Norway. We recruited the first participant group at a municipal MHD and SUD assisted living facility for service users with ongoing excessive substance use and severe social dysfunctionality. We recruited the second participant group at a community-based follow-up care service aimed at clients with a broad range of MHDs and SUDs and various levels of social functioning. The qualitative data extracted in the interviews were analyzed, using reflexive thematic analysis.

**Results:**

The analysis of the service providers’ perceptions of the barriers to social participation among clients with MHDs and SUDs revealed the following five main themes: challenging or lacking social connections, impaired cognitive functions, negative self-perception, impaired personal functioning, and insufficient social security. The barriers identified are interrelated in a cluster of cognitive, socioemotional, and functional impairments, leading to a severe and diverse complex of barriers to social participation.

**Conclusions:**

Social participation relies on people’s capability to use their present social opportunities. Promoting basic human functioning is key to promoting social participation among people with MHDs and SUDs. The findings in this study indicate a need to address cognitive functioning, socioemotional learning, instrumental skills, and complex social functions to meet the complexity and diversity of the identified barriers to social functioning in our target group. Virtual reality–based interventions for promoting social participation should be sequenced into distinct scenarios dedicated to specific learning goals to build complex learning in a step-by-step process based on successively more complex levels of human and social functioning.

## Introduction

### Background

People with mental health disorders (MHDs) and substance use disorders (SUDs) are a highly vulnerable group**,** particularly affected by social exclusion, stigmatization, disempowerment, and disconnectedness [1-3]. Many of these individuals are marginalized in terms of social participation, including in terms of education, employment, accommodation, family, friends, intimate relationships, and meaningful activities [2,4-6]. They are also at a higher risk of being survivors of domestic violence, exploitation, and abuse [2,7,8]. Recovering from MHDs and SUDs is a complex personal and social process that takes place within the context and goal of people’s lives in their community [4,9,10]. The concept of mental health recovery has been defined as “a deeply personal, unique process of changing one’s attitudes, values, feeling, goals, skills and/or roles” and “a way of living a satisfying, hopeful, and contributing life even within the limitations caused by illness” [11].

Recovery has been conceptualized as a vision, a philosophy, a process, an attitude, a life orientation, an outcome, and a set of outcomes [12]. Within clinical practice and research, a framework of 5 key recovery processes has been identified: the CHIME (connectedness, hope, identity, meaning, and empowerment) framework [12]. This framework distinguishes between a biomedical focus on “getting better” in terms of symptom reduction and a biopsychosocial focus on “recovering a life,” which entails a focus on enabling individuals to connect and interact with their social surroundings and on aiding individuals in living full and contributing lives as active citizens [13-15]. This involves finding ways to live well, either with or without symptoms or clinical problems, through a diverse, multidimensional recovery process involving the negotiation of positive social identities, supportive personal relationships, and social participation [4]. However, many lack the knowledge, skills, and capabilities to engage in their recovery.

Virtual reality technology holds the potential to create social environments and interactions that accommodate experiences needed for individuals to acquire new capabilities for the benefit of their social participation and recovery. Virtual reality–based interventions (VRIs) provide digitally simulated scenarios of real-life environments and situations that are dangerous, infrequent, or difficult to recreate in real life. VRIs allow for immediately available and greater treatment input and can reduce inconsistencies in human intervention delivery. VRIs can be graded in difficulty and endlessly repeated until the desired acquisition is achieved [16]. An emerging number of studies focusing on learning and social functioning show that VRIs represent a promising complementary measure to therapy in the treatment of a wide range of mental health conditions [17-20]. However, it is still unclear how we can harness the greater ecological validity of VRIs when targeting social functioning impairments among individuals recovering from concurrent MHDs and SUDs.

The ecological validity of VRIs may be understood as referring to how far the interventions improve functioning, daily life skills, compensatory strategies, social participation, and psychological well-being in the end users’ real-life settings and situations [17-19].

As of today, the field of virtual reality in psychiatry shows wide variation in conceptual use and methodological anchoring, with methods and concepts mixed across treatment-oriented and training-oriented simulation programs, different learning methods, and different simulation methods [21,22].

Currently, there is much to be done in understanding how we may expand the ecological validity of VRIs and create virtual reality experiences and environments that are highly relevant and realistic with regard to our target groups’ real-life problems in their real-life contexts [23]. Within the field of psychosocial or mental health VRI development, improved social functioning is the main goal [17]. This suggests a need to pin down clear operationalizations of socioemotional mechanisms and sociofunctional outcomes to obtain a deeper understanding of the factors that contribute to social participation and provide a broader understanding of how we can model socioemotional learning experiences in digital, data-rich game environments.

A clear limitation in the research on the development and assessment of psychosocial VRIs in the recent reviews is the large scope of the concept of social functioning [17]. The concept of social participation is often associated with social inclusion, which has been defined in the European Union as “a process which ensures that those at risk of poverty and social exclusion gain the opportunities and resources necessary to participate fully in economic, social and cultural life and to enjoy a standard of living and well-being that is considered normal in the society in which they live*”* [24]*.* This definition stems from traditional concerns of social science and social policy development. This is a right-based approach that is based on the assumption that social inclusion is about people’s opportunity to participate in key functions or activities in their society. This concept is closely related to the concept of citizenship, which refers to “the possession by members of a community of a range of social and cultural rights and responsibilities, by virtue of their membership of that community and as a distinct element of their national citizenship rights” [24]. However, VRIs do not change the civic rights of the end users. Therefore, these concepts of social participation are too narrow as a basis for developing VRIs.

Huxley et al [24] refer to Beck [25], who have proposed an overarching conceptual framework of social quality, defined as “The extent to which citizens are able to participate in the social and economic life of their communities, under conditions which enhance their well-being and individual potential.” This definition encompasses an understanding of promoting social participation as an empowerment process aimed at enabling people to develop their full potential in social, economic, political, and cultural processes [24]. This is in accordance with the understanding that the self-empowerment of patients is considered a necessary component in the recovery process of finding a way of social inclusion and participation in the society.

### Goal of This Study

The aim of this study was to explore how service providers in community-based MHD and SUD health care (CBHC) services perceive the barriers to social participation among adults recovering from MHDs and SUDs to provide a broader understanding of how learning experiences can be modeled to promote social participation in virtual reality environments.

This study is based on the recommendations for the methodology of virtual reality clinical trials in health care (Virtual Reality Committee of Outcomes Research Experts [VR-CORE]). This scientific framework for VRI development was established to ensure scientific rigor in the development and evaluation of virtual reality applications. VR-CORE is based on similar established standards, such as the Food and Drug Administration’s I- to III-phased model for pharmacotherapy development [26]. Within the VR-CORE framework, the VRI development phases are referred to as *VR1*, *VR2*, and *VR3*. This study is 1 of the 3 substudies in the VR1 phase of the overarching virtual reality development project “Virtual Reality (VR) as a Facilitator for Participation in Society Among Persons With Mental Health/Substance Use Disorders (MHD and SUD).” The overall goal of this project is to develop and test a new social participation VRI paradigm to promote social participation among people recovering from MHDs and SUDs.

In the VR1 phase of the VR-CORE framework, the focus is on content development by working with patient and provider end users through principles of human-centered design. The VR-CORE framework and virtual reality applications should be designed with direct input from patient and provider end users. A lack of patient involvement, poor requirement definitions, and nonadaptation to user feedback are some of the common factors that explain the failures of digital interventions. Thus, both patient and provider end users’ knowledge, attitudes, beliefs, and experiences are fundamental to developing VRIs with satisfactory ecological validity [26].

## Methods

### Design

To explore the research question of this study, we conducted 2 semistructured, open-ended focus group interviews with health care professionals in CBHC services for people recovering from MHDs and SUDs. We used reflexive thematic analysis with an inductive approach to analyze the qualitative data [27].

### Setting and Sampling

We conducted this study in a medium-sized municipality in Eastern Norway with professional service providers from CBHC services as participants. The purpose of this study required data covering a broad spectrum of social impairment severity in different stages of recovery among clients, as well as a broad array of professional perspectives within CBHC service provision.

In this context, we conceptualize CBHC services as health care services offered outside of traditional acute or specialized health services aimed at protecting, maintaining, or restoring people’s health, social functioning, and welfare within an everyday care framework. Unlike providers of specialized health services, the service providers in CBHC settings support their clients in coping with their everyday social problems in their everyday social settings. Many clients with MHDs and SUDs have extensive needs for treatment and support, and service providers often follow them up for several years. This leads to unique insights into their everyday barriers to social participation and is a highly relevant perspective for understanding our target group’s problems related to social participation.

To ensure that the focus groups covered the full range of data required to fulfill our research aim, we used theoretical sampling. This entails deliberate sampling of salient characteristics among participants, drawing upon the researchers’ prior knowledge of the topic and research aim [28]. We defined our inclusion criterion as health care professionals in CBHC with a minimum of 2 years of experience in health or social service provision to adults in various stages of recovery from MHDs and SUDs in CBHC services.

### Recruitment

We recruited professional service providers from 2 different kinds of CBHC services in our collaborating municipality. We recruited our participants through our second author, who is employed by the municipality and thus acted as a liaison between the research team and participants. One group of health care professionals came from an MHD and SUD assisted living facility, which provides housing, treatment, and care to people with ongoing excessive substance use and severe social impairments.

The other group of health care professionals was recruited from a community-based outreach follow-up service aimed at clients with a broad range of MHDs and SUDs in various stages of recovery. All participants were informed of the topic and research aim by the first and second authors before the focus group interviews. The characteristics of the participants in our sample are presented in Table 1.

**Table 1:** Participant characteristics.

Participant	Profession	Level of experience^a^
1	Peer support worker	3
2	Unskilled	3
3	Child support worker	1
4	Psychiatric nurse	1
5	Teacher	2
6	Social educator	1
7	Psychiatric nurse	4
8	Psychiatric nurse	4
9	Peer support worker	1
10	Psychiatric nurse	4
11	Social educator	4
12	Nurse	4
13	Psychiatric nurse	3
14	Teacher	3

^a^1=2 to 5 years, 2=5 to 10 years, 3=10 to 20 years, and 4=>20 years.

### Data Collection

The first and second authors conducted 2 semistructured, dual-moderator focus group interviews in June 2022. The interviews took place at the service providers’ workplaces and focused on their clients’ social challenges and how the service providers support them in coping with social functioning impairments. Semistructured focus group interviews are a well-suited data collection method for accessing practitioners’ understandings and interpretations of their practice experiences. As participants compare their experiences and negotiate their understandings during the group process, focus group interviews are better suited for providing complex formations of meaning than individual interviews [29]. Moreover, a focus group presents a more natural environment than an individual interview because participants influence and are influenced by others, just as in real life [30]. The presence of 2 moderators ensures that the focus group proceeds as planned and that all the participants interact during the session. The division of roles enables a smooth progression of the session, where all the required topics are covered [30,31]. We followed a semistructured interview guide. It was translated from Norwegian and is provided in Textbox 1.

**Interview guide.**

**Initial questions**

- What is your professional background?
- How long have you been working with people in recovery from mental health disorder (MHD) and substance use disorder (SUD)?

**Service provision**

- Please tell us how you work to support people with MHD and SUD to function socially in their everyday life?
- What works well in your practice?
- In your experience, what are the biggest challenges in your clients’ everyday lives?

**Clients’ social functioning impairments**

- How do you think that people with addiction and mental illness participate in our society?
- How do you feel that people with addiction and mental illness function socially in everyday life?
- How would you describe your clients’ connection to their family and close friends?
- What can be done to help people with MHD and SUD participate in society?
- What are the biggest obstacles to participation in society for people with MHD and SUD?
- How can health care professionals improve social participation for people with MHD and SUD?
- How do you find your clients’ prerequisites for strengthening their social skills?

### Data Analysis

The first author audio recorded both focus group interviews and transcribed them verbatim. To illuminate and understand our participants’ professional experiences, we conducted reflexive thematic analysis with an inductive approach to analyze the qualitative data.

Within all forms of thematic analysis, the theoretically based assumptions reflect how knowledge is best produced, and these are associated with *qualitative* paradigms [32]. Theoretically, Braun and Clarke [27] conceptualized this typology of thematic analysis as occupying a continuum from coding reliability to reflexive approaches, distinguished by small qualitative (“small q”) and big qualitative (“big Q”) values, as described by Kidder and Fine [33]. The reflexive end of Braun and Clark’s typology continuum is underpinned by big Q values, which typically include a conceptualization of researcher subjectivity as a resource for research and of meaning and knowledge as partial, situated, and contextual [27,33]. We used this principle of reflexive analysis by engaging collaborators with different analytical perspectives in the analytical process. The first author brought philosophical meta-theoretical assumptions and prior theoretical knowledge to the analysis. The first author’s part of the process was both to ensure scientific rigor and to convey theoretical and methodological knowledge to the other researchers. The second author is an experienced psychology specialist, who contributed to clinical perspectives and contextual questions related to clinical practice. The third author is a peer researcher with MHD and SUD lived experience, who brought an additional layer of analytical perspective when using his lived experience as background in the development of the codes and revision of the themes.

Reflexive thematic analysis comprises the following steps: (1) familiarizing oneself with the data, (2) generating initial codes, (3) searching for themes, (4) reviewing the themes, (5) refining and naming the themes, and (6) writing up the analysis [27]. In the first step, the first author listened to the focus group recordings, transcribed them verbatim, and read the transcripts carefully. In the second step, the first author coded the empirical statements and named the initial codes according to their descriptive meaning. Then, in the third step, the first author reread the transcripts and generated themes within and across the transcripts. These themes were then presented and discussed with the second and third authors. In the fourth step, the themes were reviewed and revised in collaboration with the second, third, and sixth authors. In the fifth step, the first author refined and renamed the themes in collaboration with the second and third author and then arranged the themes in a table to understand and explain their interrelationships. Finally, all authors edited the description of the analysis.

### Ethics Approval

This study did not involve patients or service users, and all data were collected from health care professionals. As this study is a part of a larger project involving service users, we conducted it in line with the Norwegian guidelines for medical and health research as well as the Declaration of Helsinki [34]. Our study was approved, pursuant to the Norwegian Health Research Act, paragraph 10, by the South-Eastern Regional Committee for Medical and Health Research Ethics (reference number: 421376) and the data protection officer of the Innlandet Hospital Trust (reference number: 18197741). The overall study was registered at ClinicalTrials.gov (reference number: NCT05653167). All the participants provided informed consent and could withdraw from the study at any time.

## Results

### Summary of Results

The findings of this study are the result of analyzing data from 2 different community services that represent 2 distinct client populations with different degrees of social impairment. Overall, these 2 populations illustrate the diversity among people recovering from MHDs and SUDs. To gain an overall understanding of what hinders our target group’s social participation, we sought to capture the full range of barriers to social participation among this group. Overall, we identified the following five main themes describing a complex cluster of interrelated barriers to social participation among people recovering from MHDs and SUDs: (1) challenging or lacking social connections, (2) impaired cognitive functions, (3) negative self-perception, (4) impaired personal functioning, (5) insufficient social security.

**Figure 1:**
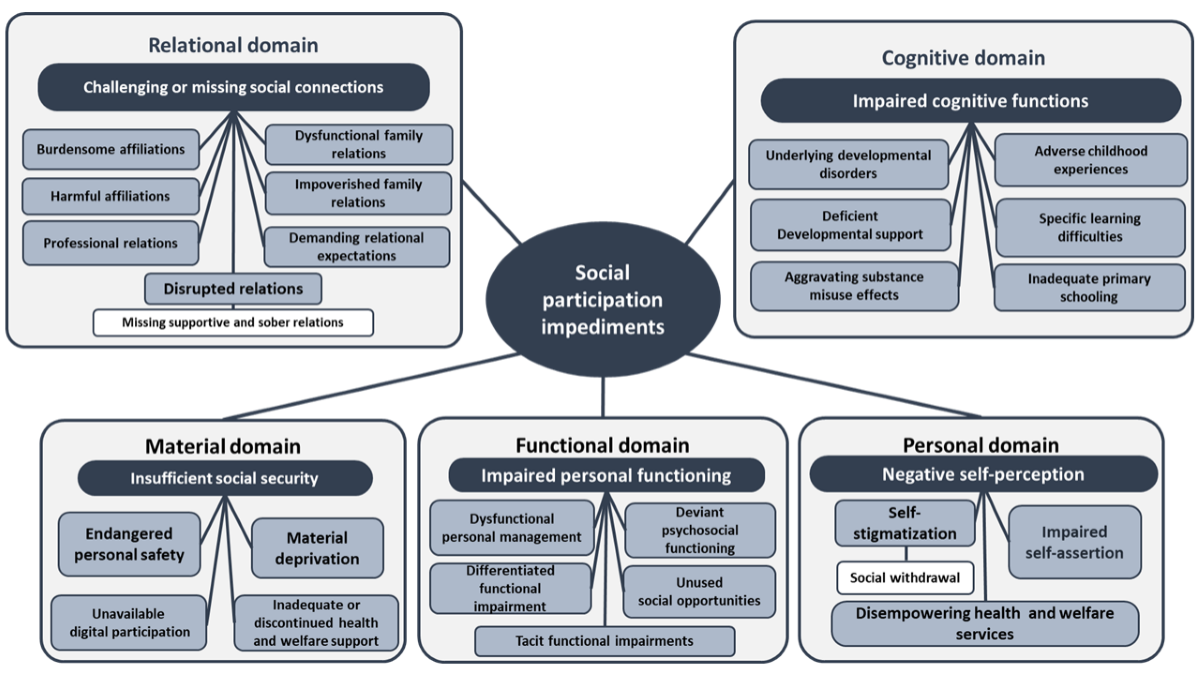
Final thematic map.

The main themes and their associated subthemes are displayed in Figure 1 in the final thematic map of our findings.

In the following sections, we present each of these themes with their subthemes and related quotes. Quotations of empirical statements are used in the description of the results to show how the content appeared in the focus group interviews. These quotations were translated from Norwegian with an attempt to preserve the original meaning as far as possible.

### Theme 1: Challenging or Lacking Social Connections

Both focus groups described social relations as a key factor for social participation. Social connectedness refers to having family, friends, and social networks to provide care, companionship, and support when needed. However, both ongoing and previous relationships may be burdensome, thus acting as barriers to social participation. For many individuals with MHDs and SUDs, their family background has led to a life with MHDs and SUDs. Some individuals with MHDs and SUDs come from traumatic family circumstances, whereas others have grown up in impoverished families:

Some have been exposed to a lot of exclusion throughout their childhood; there has been a lot of bullying, harassment and poor living conditions, your parents may have been on social security, unable to afford sports, leisure activities or school excursions, so that you fall out of your peer social network. Then it is a short path to burdensome social connections in the substance use scene, which are very inclusive

Participant 1

The focus group participants described the social connections in the drug scene as *burdensome or harmful*, where threats, violence, exploitation, and crime are common.

Burdensome or harmful social connections both sustain and reinforce social distress and thus diminish the psychological and functional prerequisites for conventional social participation.

Supportive family connections and social networks appear decisive for successful MHD and SUD recovery. However, our interviewees described how many individuals with MHDs and SUDs had *broken off their relations* with family and friends outside the drug scene:

We often start by mapping their family connections. Repairing damaged connections is often a barrier as many have badly broken relations with their family due to severe substance use and disappointments as they have stolen, exploited and done a lot of harm to their families.

Participant 1

*Broken relationships* lead to a lack of social support and companionship, and our interviewees described how many individuals with MHDs and SUDs found it very difficult to establish close relationships with “normal people.” Many individuals recovering from MHDs and SUDs try hard to avoid reconnecting with their former peers within the drug scene, as they are aware that this often leads to a relapse. Nevertheless, they often become very lonely outside their old social environment and often return to the drug scene when they become too lonely.

The participants emphasized the importance of supportive and sober relationships during MHD and SUD recovery and stated that many individuals with MHDs and SUDs greatly missed supportive companionship. However, sober and supportive relationships may also act as a burden. This perspective appeared when the interviewees discussed the burden of expectations and lack of faith among family and friends. In this way, *demanding relational expectations* lead to stress, and a lack of faith among others may diminish hope and faith in oneself during MHD and SUD recovery.

The participants also reflected on their own professional relationships with their clients, emphasizing the importance of offering real human relationships to people who are particularly affected by social disconnectedness. They also stressed the importance of genuine reciprocal relationships with their clients, as these are important for learning relational skills:

I think many have negative experiences from before, their service providers have quit or given up on them. For many, it is about trust and testing to see if we can stand them, like are you there for me for real, or are you just saying you are?

Participant 7

### Theme 2: Impaired Cognitive Functioning

The participants in both focus groups discussed how their clients are often challenged by disorders that prevent normal social functioning, in addition to hampering their ability to cooperate with their service provider and learn social skills themselves.

The interviewees expressed a clear consensus regarding *underlying development disorders* among their clients, and they discussed how cognitive impairments are rarely addressed, even though they are frequently recognized as major barriers to social participation in this group:

...Yes, I was just about to say that too, ADHD that has not been properly investigated, there are many of those people.

Participant 13

The interviewees also perceived *inadequate developmental support* to be common during their clients’ childhood and expressed clear concern about this:

We do see major deficiencies, you know, in relation to functionality, in those with poor care and a bad upbringing that had persisted until adulthood, we do see that.

Participant 8

Another negative element of childhood development that the interviewees found to be common and highly important in their clients was complex posttraumatic disorders due to adverse childhood experiences.

Many clients seemed to have experienced severe trauma during their childhood and adolescence. In addition, many had experienced emotional and physical neglect, a lack of basic care, a dysfunctional home situation, and other kinds of accumulated traumatic distress:

He has been bullied throughout his entire schooling, all because he seems a little different.

Participant 11

The interviewees also discussed how adverse childhood experiences reduced their clients’ functioning in terms of concentration, depression, social anxiety, and trust.

Another theme discussed by the participants was that many of their clients struggle with cooperation with public services owing to poor understanding of messages and information. The service providers shared the perception that many individuals with MHDs and SUDs have *specific learning difficulties* that prevent them from performing certain types of skills or completing tasks if left to figure things out by themselves or if taught in a conventional way*:*

Dyslexia and dyscalculia, which have never been investigated...Many who can’t cope with school have learning difficulties that aren’t discovered until their regular schooling is almost over.

Participant 12

The interviewees said that many of their clients faced challenges with reading and writing, and some of them were described as illiterate. This was particularly evident among immigrant clients with little education in their mother tongue and Norwegian.

Moreover, the participants found that many of their clients had *inadequate elementary schooling,* as many had often skipped classes, dropped out of school very early, or regularly been taken out of their class and given alternative work:

In my experience, so many of my clients hung out a lot with the janitor at school. They were sent out of their classes to hang out with the janitor, and eventually the janitor became a safe adult caregiver for them.

Participant 13

For many, this not only led to poor academic skills but also affected social functioning in many ways, as they missed both academic and social learning when dismissed from class. An example of this discussed in the focus groups is that a lack of common social knowledge about one’s community and society impedes social participation, as without common social knowledge, one struggles to understand and keep up with what people are interested in during everyday small talk about current topics. The participants felt that this contributed to the avoidance of social participation for many clients.

The final theme regarding the prerequisites for social functioning was the implications of substance use:

But in relation to learning abilities as well as other functioning problems, I think many of those within the drug scene, who have been through heavy substance use, have major problems with their memory to a greater or lesser extent, and that this may be due to excessive intoxication and overdoses.

Participant 12

The participants in both focus groups talked about how they found a connection between poor social functioning and substance use, which illustrated the *aggravating effects of substance abuse.*

### Theme 3: Negative Self-perception

The service providers discussed several points regarding their clients’ levels of self-assertion, one of which was *negative self-perception*:

They are so focused on their self-perceived lack of skills, they may possess the skills, but they do not believe in themselves, so they start off with an assumption that everything is completely hopeless.

Participant 10

The interviewees discussed how many of their clients often appeared evasive toward others, demonstrating a very negative self-perception.

The participants also described *self-stigma* as a key mechanism for low social participation, which also prevented clients’ *self-assertion*:

In a way it is a lot about them stigmatizing themselves more than others around them do.

Participant 1

Being self-assertive is about standing up for one’s own wishes, preferences, and personal agenda and is key to using social opportunities. Therefore, impaired self-assertion acts as a key barrier to social participation. For many, this leads to *social withdrawal*, at least from what would be considered conventional social settings, as they keep to themselves because of their self-stigma:

Then he just says I don’t have proper clothes or don’t look good enough to be with other people at an event like that, so he ends up not going.

Participant 1

The interviewees discussed their own impact on their clients and reflected on how they assist their clients in their recovery. A recurrent theme throughout this discussion was how many clients had negative experiences with *disempowering health and social services:*

There are a lot of top-down attitudes in the system, in my experience. And when you have experienced that a few times, it is very easy to get defensive.

Participant 10

The interviewees also discussed their own practice and talked about how helping too much can hamper their clients’ recovery:

We don’t help our clients by doing things for them all the time, you know. I think accountability is an important word when supporting people.

Participant 14

Excessive help thus acts as a mechanism that disempowers clients instead of teaching them the skills they need to become independent; in this case, the outcome seems to be a kind of *unachieved recovery*, where clients achieve sobriety but do not engage in conventional social participation, and their recovery seems to stall at some point.

### Theme 4: Impaired Personal Functioning

The participants in both focus groups discussed at length their clients’ personal functioning, or the lack thereof, in their daily lives. We conceptualized this theme as impaired personal functioning; personal functioning refers to a person’s physical, psychological, and social capabilities for coping with everyday activities they find meaningful and necessary.

*Impaired functional competence* is a major challenge for many individuals with MHDs and SUDs, according to the interviewees. Functional competence involves capabilities of basic and instrumental daily functioning, such as maintenance of personal hygiene, household management, shopping, cooking, financial management, and health management:

...not to mention going to the grocery store! They have never actually shopped for groceries before, so they just wander about the store not understanding where anything is placed, so it is no wonder they just buy unhealthy food, because they just grab whatever is closest to the checkout.

Participant 13

In the discussions about client functioning, the interviewees described many ways in which *deviant psychosocial functioning* was expressed through behaviors such as messing up and destroying apartments:

We have for example clients who have lived in many different places, who have destroyed several of their apartments worth millions.

Participant 2

The interviewees described great variation in their clients’ levels of functioning. Nevertheless, many struggle to understand and cope with what is considered normal social behavior in complex situations with particular expectations of such behavior, such as meetings, courses, and cultural events. Many of those in greatest need of care were caught up in substance use in early adolescence and have missed out on many aspects of socioemotional development:

If you start getting high at the age of twelve, you miss out a lot.

Participant 12

The participants felt that social functioning before substance use onset was crucial for client functioning after achieving sobriety:

It could be about living a normal life prior to the substance use onset, if the substance use has not come too soon. Then they may be able to get on, because they have learned social skills and household management and all of that already.

Participant 7

Some were caught up in substance use in their twenties and had completed compulsory schooling and vocational education, which led to social opportunities that they did not use. During the discussions on several topics, the interviewees described how their clients rarely spoke explicitly about social impairments such as poor reading and writing skills and how many relied on their family or peers for help.

Often, social functioning impairments first appear in behaviors applied to daily activities, much like tacit knowledge that appears through practice applied in clinical settings during observation of professional service providers:

I thought he had social anxiety about going on that bus, so we started working on that, but then it appears the problem is something completely different, that he can’t read the bus timetable. He doesn’t understand the bus timetable, which is why he does not show up for our appointments.

Participant 2

This indicates that one may relate social functioning impairments to tacit functioning impairments in the same way that one may relate social knowledge to tacit knowledge.

### Theme 5: Insufficient Social Security

Improving adverse living conditions was a topic of great concern among the participants in both focus groups. Insufficient social security refers to having insufficient access to food, shelter, personal hygiene, clothing, basic health services, and an appropriate living standard. This emphasizes clients’ dependence on safe accommodation and housing support owing to functioning impairments as well as MHDs and SUDs.

In the assisted living facility, they experienced the impact of personal safety during ongoing excessive substance use:

They still use substances excessively, but there is a lot less crime, there is less violence and there is a great change regarding threats, weapons and all of that, compared to when they live out there.

Participant 2

This illustrates both the importance of assisted living facilities and the level of personal risk and danger clients are exposed to when living on skid row or in unstaffed social housing in the company of other substance abusers. These risks include violence, robbery, exploitation, and abuse. Such *dangerous living condition*s thus lead to severe social distress and are contradictory to the recovery process.

*Financial management* was also a key topic in both focus groups. Here, the interviewees were particularly concerned about an increasing number of services, including financial support, being available only on the web, thus becoming increasingly unavailable for many people. For clients with MHDs and SUDs, a *lack of internet skills* hinders their social participation, as their access to digital services is limited:

Very few of our clients have online skills, so digital services are a big obstacle for them. They should be able to take responsibility for this themselves, but they may only have an old prepaid phone without any smartphone features, they don’t have access to a computer or tablet, some do, but far from all, and what’s more, it also depends on their ability to use it.

Participant 7

Furthermore, this illustrates the importance of continuity and efficacy in health and social services and indicates that *inadequate or discontinued health and social support* is a key barrier to social participation for people with MHDs and SUDs, as these services also provide safety and important protection from *material deprivation*.

## Discussion

### Principal Findings

The purpose of this study was to explore CBHC service providers’ perceptions of the barriers to social participation among people recovering from MHDs and SUDs. Overall, this study suggests that social participation relies on basic human functioning within social, cultural, and societal contexts. These findings imply that impaired personal functioning, which leads to self-stigma and social withdrawal, is a key theme in our study (Figure 1). The findings describe how social and functional impairments in clients with MHDs and SUDs are associated with a prominent presence of neurocognitive impairments. The service providers’ descriptions of the early onset of both psychosocial and substance use problems among many of their clients indicate that barriers to social participation may be associated with underlying neurodevelopmental disorders as well as MHDs.

These findings concur with previous results from studies of concurrent SUDs and autism spectrum disorders and studies of concurrent SUDs and mild to borderline intellectual disability [35,36]. This is also in line with studies of mild to borderline intellectual disability in a Norwegian polysubstance use cohort, which showed that borderline intellectual functioning is more common among persons with MHDs and SUDs than in the general population [37]. Developmental disorders were found to exist before substance use in this population [37-39]. Patients with MHDs and SUDs are rarely assessed for developmental disorders, despite severe functional impairment [35]. Concurrent developmental disorders, such as autism spectrum disorders in combination with SUDs and mild to borderline intellectual disability in combination with SUDs, are overlooked and scarcely addressed in the field of adult MHDs and SUDs [35,36].

Neurodevelopmental disabilities are a group of conditions due to impairments in physical, learning, language, or behavioral areas. These conditions include, but are not restricted to, attention-deficit/hyperactivity disorder; autism spectrum disorders; intellectual disabilities; Tourette syndrome; and a wide range of metabolic, genetic, and hereditary disorders. All these are brain conditions that affect children’s cognitive development as they grow and develop necessary life skills; this has a subsequent impact on basic functioning throughout the children’s life span [40]. Cognitive functions refer in essence to all kinds of complex brain activities and mental abilities, such as learning, thinking, reasoning, remembering, understanding, problem solving, and decision-making. Various forms of cognitive impairments hinder social participation and social learning capability [41].

Cognitive impairments represent a potential added risk factor for elevated psychological distress, detrimental treatment outcomes, and relapse in populations with MHDs and SUDs [37-39,42]. Further investigation has revealed that patients with cognitive impairments have lower educational levels, greater self-reported childhood learning difficulties, more history of previous contact with support systems, and more instances of substance use relapse during treatment than their peers without cognitive impairments [39].

Many children affected by developmental disorders learn to cope with their challenges and live fulfilled happy lives as adults; however, these children are far more dependent on adequate developmental support than others. Hence, individuals affected by developmental disorders are much more susceptible to care failure, distress, and adverse childhood events than others.

Our findings also suggest a greater presence of adverse childhood experiences and dysfunctional upbringing among persons with MHDs and SUDs than in the general population. This is in line with a number of studies of both MHDs and SUDs and posttraumatic stress disorder in adult and adolescent populations [8]. Adverse childhood events are events experienced by a child that evoke fear and are commonly violent, dangerous, or life-threatening. Physical, sexual, and psychological abuses are typically referred to as traumatic or adverse childhood experiences. However, psychosocial distress that, for adults, would be tolerable may lead to severe posttraumatic stress disorders when experienced by children and adolescents. For children, this also involves emotional abuse and neglect, physical neglect, and dysfunctional home situations, such as where the child witnesses domestic violence [43]. Both dysfunctional upbringing and prior adverse childhood events are immutable factors that disturb the affected individual’s cognitive functioning throughout their life span [44]. Children exposed to adverse experiences or with accumulated traumatic stress often develop severe impairments affect and bodily regulation, attention and behavior regulation, and socioemotional regulation [44]. It has also been found that childhood traumatization results in more serious disorders with poorer mental and physical health, more polysubstance use, a higher degree of drug addiction, poorer levels of work, poorer work capacity, and poorer treatment outcomes than traumatization in adults [45].

This study also showed that insufficient elementary schooling may act as an obstacle to adult social participation. The acquisition of socioemotional skills is an essential developmental process through which children learn to act and behave appropriately in social interactions and to form and maintain healthy relationships with others as adults [46]. Social skills development is promoted both in domestic environments and at school. Academic achievement and social interaction with peers in class, particularly during middle school, are crucial for acquiring social skills. A well-led and orderly classroom not only is conducive to academic learning but also models appropriate behavior and communicates rules for acceptable and positive behavior. Hence, early school dropout or excessive classroom dismissal robs certain children of opportunities to practice academic and socioemotional skills [46]. This study demonstrates how this affects pupils’ everyday social functioning as adults and shows that academic skills are also key to promoting social participation.

Moreover, this study indicated an association between substance abuse and social functioning, which is in line with several studies on this topic [8]. Acute substance use effects, adverse substance use consequences, and various withdrawal symptoms are likely to aggravate already impaired social functioning.

Substance use affects a number of neurophysiological mechanisms, including inhibitory behavioral mechanisms in the frontal lobe and the levels of noradrenaline and serotonin in the limbic structures. This disturbs the cognitive capacity to regulate impulsive actions and emotional reactions, which, in turn, leads to impaired judgment, hyperactivity, cognitive disturbances, emotional dysregulation, and increased aggression [47].

Overall, this study showed that cognitive and functional impairments may act as key barriers to social participation. This is in line with the conceptualization of social participation in the study by Huxley et al [24], where social participation was conceptualized as a function of people’s available social opportunities in the society they live in and people’s capability to use their available social opportunities. This places basic human functioning at the root of social participation. This indicates that basic human functioning may act as a key learning goal for VRIs dedicated to promoting social participation.

Basic human functioning is commonly viewed as a wide range of complexities required for functioning in a diverse range of everyday tasks [41]. The lowest level of human functioning is called life maintenance, which is followed by successively more complex levels of functioning, namely *perception-cognition, physical self-maintenance, instrumental self-maintenance, effectance and social behavior*. Each of these levels of functioning generally requires a greater complexity of neuropsychological organization than the preceding level [41].

Effectance is in essence the driving force behind individuals’ social participation. The term effectance is conceptualized as the human motivation to proactively participate in and interact with one’s social environments. In this sense, social participation, including participation in terms of education, employment, accommodation, family, friends, intimate relationships, and meaningful activities, may be understood as the practical outcome of individuals’ effectance. The result of people’s effectance is linked to a sense of autonomy; competence self-determination; and, ultimately, quality of life [48,49]. People’s effectance relies on their perception of personal resources, such as the knowledge, skills, and abilities needed to explore and master our social environment [48,49]. This perception of personal resources and competencies for exploring and mastering various social settings and situations builds upon one’s capability to perform activities required to maintain adequate basic self-care and uphold day-to-day personal security and basic quality of life. This is referred to as *instrumental self-maintenance* [41].

Instrumental self-maintenance consists of the functions that enable individuals to take independent responsibility for their basic safety and security in their daily living. This involves, for example, grocery shopping, cooking, household management, financial management, health management, psychosocial maintenance, and emergency maintenance [41]. All these capabilities rely heavily on *socioemotional abilities*, such as the ability to accurately recognize one’s emotions and thoughts and their influence on behavior and the ability to regulate one’s emotions, thoughts, and behaviors in different situations, including situations warranting stress management, adaptation, time management, self-monitoring, and self-regulation. Socioemotional functions also involve the ability to establish and maintain healthy and rewarding relationships with diverse individuals and groups [50-52].

Socioemotional abilities also include the abilities to take a perspective, empathize with others from diverse backgrounds and cultures, understand social and ethical norms for behavior, resist inappropriate social pressure, and set boundaries for others. Other socioemotional abilities are responsible decision-making skills, such as making constructive and respectful choices about one’s personal behavior and social interactions based on ethical standards, safety concerns, and social norms [53]. The performance of socioemotional skills, in turn, relies on *executive functions* [50,51,53,54]. Executive functions are conceptualized as a single function through which individuals gain knowledge and solve problems. This function involves 9 different but interconnected capacities: attention, emotion, regulation, flexibility, planning, organization, initiation, inhibitory control, and working memory [54].

### Practical Implications for VRI Development

A clear understanding of the learning content needed to facilitate the acquisition of basic human functioning and social skills in VRIs is essential for effectively promoting social participation in real-life settings. Overall, the results of this study suggest that the efficient promotion of social participation requires a diverse VRI paradigm that accommodates cognitive remediation, socioemotional learning, instrumental skills training, and complex social functioning exercises. In addition, similar to all types of learning activities, VRIs must be designed, structured, and sequenced carefully in a deliberate workflow to effectively promote the desired learning outcomes [55]. This indicates that VRIs should be sequenced into distinct scenarios dedicated to specific learning goals to build complex learning in a step-by-step process based on successively more complex levels of human and social functioning.

The indication in this study of a high incidence of cognitive impairments and specific learning disabilities among people with MHDs and SUDs not only provides information about the necessary learning content for the VRI paradigm but also underlines the need for a comprehensive understanding of how the VRI end users acquire the capabilities needed to achieve the desired outcomes of the VRI paradigm. The concept of acquisition refers to how people acquire abilities and construct knowledge through mental information; how cognitive structures develop and change; and how repertoires of new behaviors are acquired, used as practical intelligence, and applied in strategies or capacities to cope with challenging or new situations in real-life settings [56]. This entails the need for a detailed examination of how embodied, social, and spatial virtual environments affect the learning mechanisms of VRIs [57,58]. Hence, the specific affordances of the virtual reality media and factors that influence the acquisition of social capabilities should be considered when designing VRIs [59].

Cognitive remediation, socioemotional learning, instrumental skills training, and complex social functioning rely on different neuropsychological capabilities that individuals acquire through different learning mechanisms. Thus, the acquisition of cognitive functions, practical skills, and social behavior is based on various learning mechanisms that require different methodological approaches to facilitating learning. VRIs also include substantial learning complexity, with a wide variety of learning mechanisms affecting participants’ learning acquisition and the ecological validity of the VRIs [21,60-62]. The main challenge of expanding the ecological validity of VRIs is found in the human limitations in transferring skills from virtual to real-life environments [63-65]. In VRIs, the entire virtual environment is artificially generated, which, therefore, also applies to all the learning mechanisms affecting the participants’ learning. The ecological validity of VRIs is, therefore, dependent on purposeful and accurate learning conveyance set in virtual environments with satisfactory similarity to the end users’ societal and cultural real-life environments [59,65,66].

Mitigating violence, exploitation, human abuse, and social marginalization is crucial for people’s engagement in social participation and recovery [7,67,68]. Social distress and disparities are not affected by VRIs dedicated to clients with MHDs and SUDs and need to be addressed through political and civic measures. Nevertheless, maintaining an emancipatory perspective is always important when addressing problems regarding MHDs and SUDs.

### Strengths and Limitations

This study includes data from 2 focus group interviews with a total of 14 participants—6 (43%) contestants in one and 8 (57%) contestants in another—which provided valuable insights into the complex relationships among MHDs and SUDs, social functioning, and social participation in a group of persons particularly affected by social distress, marginalization, and disconnectedness. A strength of this study is the use of focus groups, which promote reflection and encourage the participants to be linguistically explicit as they discuss the topics involved. Focus groups are particularly well-suited for eliciting rich data when they consist of professionals with the necessary experience and linguistic skills to understand and clearly describe their experiences and views on their clinical practice. Another strength is the theoretical sampling of participants from CBHC services who had followed their clients’ daily lives for extended periods. This provided intimate insights into their clients’ everyday struggles. The spectrum of different professions with varied perspectives in the focus groups enhanced the discussions and added nuances and depth to our data.

In our reflexive thematic analysis, the themes were revised with a peer support researcher and a specialist psychologist, who were also involved in discussions on the understanding of how the themes were interconnected. This deepened our understanding of the findings of this study and their further application.

This study also has some limitations. People with MHDs and SUDs comprise a multifaceted group of individuals with a wide range of disparities. We did not exhaustively investigate social participation or recovery, as this study was limited by its small sample size and narrow scope. Consequently, this study does not represent all individuals with MHDs and SUDs. Its focus was on understanding the barriers to social participation in the context of developing VRIs to promote social participation among people recovering from MHDs and SUDs. Hence, this study was purposively designed to provide actionable results to inform the further development and use of VRIs.

As the focus groups only included participants from the same health care services, personal relations and group dynamics may have influenced the consensus and dissention. The participants’ experiences represented only local practices. Moreover, this study was conducted in Norway, one of the northern welfare states in which using social opportunities and civic rights will be more relevant than in countries with other welfare systems.

### Conclusions

This study illustrates how a complex cluster of neuropsychological capabilities forms the basis of individuals’ social participation. Basic human functioning seems essential for promoting instrumental functioning and enabling people to connect and interact with their social environment and use the social opportunities in their communities. This is scarcely addressed in MHD and SUD rehabilitation and is not targeted in the development of VRIs aimed at people recovering from MHDs and SUDs [69]. The present empirical and theoretical material indicates a need for diverse VRI applications that accommodate cognitive remediation, socioemotional learning, instrumental skills training, and complex social functioning exercises to promote social participation among people recovering from MHDs and SUDs, people with autism spectrum disorders in combination with SUDs, and people with mild to borderline intellectual disability in combination with SUDs.

The application of a didactic theoretical framework to VRI development will also provide essential information about the specific ecological outcomes of the VRIs. This is key to determining the affordance and efficacy of the VRIs. Moreover, this will make the VRIs suitable for investigating neurocognitive impairments and enable the assessment of functional progress among the participants. This is an important step on the path to understanding how we can develop VRIs that provide the skills, abilities, and functions that people recovering from MHDs and SUDs need to cope with social impairments and engage in social participation in their societies.

## Abbreviations

CBHC: community-based mental health disorder and substance use disorder health care
CHIME: connectedness, hope, identity, meaning, and empowerment
MHD: mental health disorder
SUD: substance use disorder
VR-CORE: Virtual Reality Committee of Outcomes Research Experts
VRI: virtual reality–based intervention

## Data Availability

The anonymized transcripts used in this study are available from the corresponding author upon request.

## References

[1] Boardman J, Killaspy H, Mezey G (2022). Social Inclusion and Mental Health.

[2] Hustvedt I, Bosnic H, Emmerhoff M (2021). Utredning Om Fact Ung I Norge.

[3] Freeman D, Reeve S, Robinson A, Ehlers A, Clark D, Spanlang B, Slater M (2017). Virtual reality in the assessment, understanding, and treatment of mental health disorders. Psychol Med.

[4] Sommer M, Biong S, Borg M, Karlsson B, Klevan T, Ness O, Nesse L, Oute J, Sundet R, Kim HS (2021). Part II: living life: a meta-synthesis exploring recovery as processual experiences. Int J Environ Res Public Health.

[5] Tew J, Ramon S, Slade M, Bird V, Melton J, Le Boutillier C (2011). Social factors and recovery from mental health difficulties: a review of the evidence. Brit J Social Work.

[6] Lie T, Hustvedt IB (2021). Personer med ROP-lidelser og alvorlige psykiske helseproblemer – tjenestemottakere med store helse‑ og levekårsproblemer. Tidsskrift psykisk helsearbeid.

[7] Luchenski S, Maguire N, Aldridge RW, Hayward A, Story A, Perri P, Withers J, Clint S, Fitzpatrick S, Hewett N (2018). What works in inclusion health: overview of effective interventions for marginalised and excluded populations. Lancet.

[8] Aasen J, Lien L, Lie TW (2022). Møter med vold og suicidalitet. Sammensatte problemer, sammenvevde tiltak: Integrert behandling av rus og psykiske lidelser.

[9] Rowe M, Davidson L (2016). Recovering citizenship. Isr J Psychiatry Relat Sci.

[10] Klevan T, Bank R, Borg M, Karlsson B, Krane V, Ogundipe E, Semb R, Sommer M, Sundet R, Sælør KT, Tønnessen SH, Kim HS (2021). Part I: dynamics of recovery: a meta-synthesis exploring the nature of mental health and substance abuse recovery. Int J Environ Res Public Health.

[11] Anthony WA (1993). Recovery from mental illness: the guiding vision of the mental health service system in the 1990s. Psychosocial Rehab J.

[12] Leamy M, Bird V, Le Boutillier C, Williams J, Slade M (2011). Conceptual framework for personal recovery in mental health: systematic review and narrative synthesis. Br J Psychiatry.

[13] Whitley R, Drake RE (2010). Recovery: a dimensional approach. Psychiatr Serv.

[14] Sommer M, Finlay L, Ness O, Borg M, Blank A (2019). “Nourishing communion”: a less recognized dimension of support for young persons facing mental health challenges?. Humanistic Psychologist.

[15] Slade M, Amering M, Farkas M, Hamilton B, O'Hagan M, Panther G, Perkins R, Shepherd G, Tse S, Whitley R (2014). Uses and abuses of recovery: implementing recovery-oriented practices in mental health systems. World Psychiatry.

[16] Cieślik B, Mazurek J, Rutkowski S, Kiper P, Turolla A, Szczepańska-Gieracha J (2020). Virtual reality in psychiatric disorders: a systematic review of reviews. Complement Ther Med.

[17] Riches S, Pisani S, Bird L, Rus-Calafell M, Garety P, Valmaggia L (2021). Virtual reality-based assessment and treatment of social functioning impairments in psychosis: a systematic review. Int Rev Psychiatry.

[18] Schroeder AH, Bogie BJ, Rahman TT, Thérond A, Matheson H, Guimond S (2022). Feasibility and efficacy of virtual reality interventions to improve psychosocial functioning in psychosis: systematic review. JMIR Ment Health.

[19] Jahn FS, Skovbye M, Obenhausen K, Jespersen AE, Miskowiak KW (2021). Cognitive training with fully immersive virtual reality in patients with neurological and psychiatric disorders: a systematic review of randomized controlled trials. Psychiatry Res.

[20] Meyerbröker K (2021). Virtual reality in clinical practice. Clin Psychol Psychother.

[21] Mitra P, Fluyau D (2014). The current role of medical simulation in psychiatry. Treasure Island.

[22] Gerup J, Soerensen CB, Dieckmann P (2020). Augmented reality and mixed reality for healthcare education beyond surgery: an integrative review. Int J Med Educ.

[23] Scavarelli A, Arya A, Teather RJ (2020). Virtual reality and augmented reality in social learning spaces: a literature review. Virtual Reality.

[24] Huxley P, Evans S, Madge S, Webber M, Burchardt T, McDaid D, Knapp M (2012). Development of a social inclusion index to capture subjective and objective life domains (Phase II): psychometric development study. Health Technol Assess.

[25] Beck W (1981). Social quality. Social Quality Theory.

[26] Birckhead B, Khalil C, Liu X, Conovitz S, Rizzo A, Danovitch I, Bullock K, Spiegel B (2019). Recommendations for methodology of virtual reality clinical trials in health care by an international working group: iterative study. JMIR Ment Health.

[27] Braun V, Clarke V (2020). Can I use TA? Should I use TA? Should I not use TA? Comparing reflexive thematic analysis and other pattern-based qualitative analytic approaches. Couns Psychother Res.

[28] Powell R A, Single H M (1996). Focus groups. Int J Qual Health Care.

[29] Halkier B (2010). Fokusgrupper: Den fokuserede socialitet. Kvalitative metoder : En grundbog.

[30] Krueger R (1988). Focus Groups A Practical Guide for Applied Research.

[31] O.Nyumba T, Wilson K, Derrick CJ, Mukherjee N (2018). The use of focus group discussion methodology: insights from two decades of application in conservation. Methods Ecol Evol.

[32] Braun V, Clarke V (2022). Conceptual and design thinking for thematic analysis. Qual Psychol.

[33] Kidder L, Fine M (1987). Qualitative and quantitative methods: when stories converge. New Directions Program Eval.

[34] World Medical Association (2013). World Medical Association Declaration of Helsinki: ethical principles for medical research involving human subjects. JAMA.

[35] van Duijvenbode N, VanDerNagel J (2019). A systematic review of substance use (disorder) in individuals with mild to borderline intellectual disability. Eur Addict Res.

[36] Kunreuther E (2021). Autism spectrum disorder and substance use disorder: a dual diagnosis hiding in plain sight. Psychiatr Clin North Am.

[37] Braatveit KJ, Torsheim T, Hove O (2018). The prevalence and characteristics of intellectual and borderline intellectual disabilities in a sample of inpatients with substance use disorders: preliminary clinical results. J Mental Health Res Intellect Disab.

[38] Hetland J, Braatveit KJ, Hagen E, Lundervold AJ, Erga AH (2021). Prevalence and characteristics of borderline intellectual functioning in a cohort of patients with polysubstance use disorder. Front Psychiatry.

[39] Braatveit K (2018). Intellectual Disability Among In-patients With Substance Use Disorders.

[40] Holm VA (1989). Developmental disabilities: delivery of medical care for children and adults. JAMA.

[41] Lawton MP, Brody EM (1969). Assessment of older people: self-maintaining and instrumental activities of daily living. Gerontol.

[42] Braarud HC (2012). Kunnskap om små barns utvikling med tanke på kompenserende tiltak iverksatt av barnevernet. Tidsskriftet Norges Barnevern.

[43] Wu NS, Schairer LC, Dellor E, Grella C (2010). Childhood trauma and health outcomes in adults with comorbid substance abuse and mental health disorders. Addict Behav.

[44] Cloitre M, Stolbach BC, Herman JL, van der Kolk B, Pynoos R, Wang J, Petkova E (2009). A developmental approach to complex PTSD: childhood and adult cumulative trauma as predictors of symptom complexity. J Trauma Stress.

[45] Farrugia PL, Mills KL, Barrett E, Back SE, Teesson M, Baker A, Sannibale C, Hopwood S, Rosenfeld J, Merz S, Brady KT (2011). Childhood trauma among individuals with co-morbid substance use and post traumatic stress disorder. Ment Health Subst Use.

[46] Sørlie M, Hagen KA, Nordahl KB (2020). Development of social skills during middle childhood: growth trajectories and school-related predictors. Int J School Educ Psychol.

[47] Schifano F, Zangani C, Chiappini S, Guirguis A, Bonaccorso S, Corkery J (2020). Substance-use disorders and violence. Violence and Mental Disorders.

[48] Gardner DG, Pierce JL, Lv F (2022). An empirical examination of the genesis of psychological ownership. Merits.

[49] Deci E, Ryan R (1975). Intrinsic Motivation and Self-Determination in Human Behavior.

[50] Duncan AW, Bishop SL (2015). Understanding the gap between cognitive abilities and daily living skills in adolescents with autism spectrum disorders with average intelligence. Autism.

[51] Pugliese CE, Anthony L, Strang JF, Dudley K, Wallace GL, Kenworthy L (2015). Increasing adaptive behavior skill deficits from childhood to adolescence in autism spectrum disorder: role of executive function. J Autism Dev Disord.

[52] Bell I, Pot-Kolder RM, Wood SJ, Nelson B, Acevedo N, Stainton A, Nicol K, Kean J, Bryce S, Bartholomeusz CF, Watson A, Schwartz O, Daglas-Georgiou R, Walton CC, Martin D, Simmons M, Zbukvic I, Thompson A, Nicholas J, Alvarez-Jimenez M, Allott K (2022). Digital technology for addressing cognitive impairment in recent-onset psychosis: a perspective. Schizophr Res Cogn.

[53] Gresham F, Elliott S, Metallo S, Byrd S, Wilson E, Erickson M, Cassidy K, Altman R (2018). Psychometric fundamentals of the social skills improvement system: social–emotional learning edition rating forms. Assessment Effective Intervent.

[54] Goldstein S, Naglieri J (2013). Handbook of Executive Functioning.

[55] Britain S (2004). A review of learning design: concept, specifications and tools. A report for the JISC E-learning Pedagogy Programme. JISC.

[56] Fenwick T, Tennant M (2004). Understanding adult learners. Dimensions of Adult Learning Adult Education and Training in a Global Era.

[57] Radianti J, Majchrzak TA, Fromm J, Wohlgenannt I (2020). A systematic review of immersive virtual reality applications for higher education: design elements, lessons learned, and research agenda. Comput Educ.

[58] Riches S, Bird L, Chan N, Garety P, Rus-Calafell M, Valmaggia L (2020). Subjective experience of paranoid ideation in a virtual reality social environment: a mixed methods cross-sectional study. Clin Psychol Psychother.

[59] Makransky G, Lilleholt L (2018). A structural equation modeling investigation of the emotional value of immersive virtual reality in education. Educ Tech Res Dev.

[60] Bowe SN, Johnson K, Puscas L (2017). Facilitation and debriefing in simulation education. Otolaryngol Clin North Am.

[61] Zigmont JJ, Kappus LJ, Sudikoff SN (2011). Theoretical foundations of learning through simulation. Semin Perinatol.

[62] Piot M, Attoe C, Billon G, Cross S, Rethans J, Falissard B (2021). Simulation training in psychiatry for medical education: a review. Front Psychiatry.

[63] Mann S, Furness T, Yuan Y, Lorio J, Wang Z (2018). All reality: virtual, augmented, mixed (x), mediated (x,y), and multimediated reality. arXiv.

[64] Piot M, Dechartres A, Guerrier G, Lemogne C, Layat-Burn C, Falissard B, Tesniere A (2018). Effectiveness of simulation in psychiatry for initial and continuing training of healthcare professionals: protocol for a systematic review. BMJ Open.

[65] Kaplan AD, Cruit J, Endsley M, Beers SM, Sawyer BD, Hancock PA (2021). The effects of virtual reality, augmented reality, and mixed reality as training enhancement methods: a meta-analysis. Hum Factors.

[66] Sharma A (2020). Persuasive/Serious Games: Agents of Change in Individual Beliefs.

[67] Hestevik CH, Steiro A, Smedslund G, Harboe I (2020). Norsk forskning om forebyggende tiltak og hjelpetiltak mot vold i nære relasjoner: Et forskningskart.

[68] Øverlien C (2016). Barn, vold og traumer. Møter med unge i utsatte livssituasjoner.

[69] Limberger J, Andretta I (2018). Social skills training for drug users under treatment: a pilot study with follow-up. Psicol Reflex Crit.

